# A review on periodontal care: Challenges among geriatric and pediatric patients

**DOI:** 10.6026/9732063002001532

**Published:** 2024-11-30

**Authors:** Pragya G., Anitha Logaranjani K., Prashanthi P., Vijayalakshmi R., Jaideep Mahendra

**Affiliations:** 1 Department of Periodontics, Meenakshi Ammal Dental College and Hospital, Chennai, Tamil nadu, India

**Keywords:** Geriatric, pediatric, periodontitis, periodontal care

## Abstract

Periodontal care for geriatric and pediatric patients poses distinct challenges due to age-specific factors. In elderly patients, systemic conditions, medications and reduced dexterity complicate treatment. Pediatric patients face unique issues related to developing dentition and behaviour management. This review reports these challenges and offers strategies for effective, tailored periodontal care. Emphasizing patient-centered approaches improves outcomes for both populations.

## Background:

Periodontal health is a significant concern across various age groups, presenting unique challenges for both geriatric and pediatric populations. The global aging population is expanding rapidly, with more than six hundred million people above sixty and older, projected to double by 2025 and reach 2 billion by 2050, with 80% residing in developing countries. Poor oral health among the elderly has become a pressing public health issue worldwide [1]. A critical challenge is the scarcity of epidemiological data, which hinders effective oral health care planning. In Asia, high prevalence rates of periodontitis have been observed, partly due to early studies that focused on gingivitis and moderate periodontitis attributed to poor oral hygiene and calculus deposition. Asian individuals exhibit the third-highest prevalence of periodontitis globally [2]. India exemplifies this demographic shift, with its elderly population expected to increase from 100 million to 323 million by 2050 [3]. This rapid aging trend brings numerous health challenges, including multimorbidity, malnutrition and inadequate social and public health support. These factors contribute to increased disability among the elderly, leading to dependence on others for mobility or institutionalization. Notably, India's aging population is growing and is characterized by a higher proportion of elderly women [4]. Oral health is intricately linked to overall health, happiness and well-being, with periodontal disease severity generally increasing with age due to cumulative effects and declining immune function [5]. Conversely, dentistry addresses the oral health needs of infants, children and adolescents, aiming to establish lifelong oral health habits and prevent future dental problems and may affect the peri-odontium. Early intervention and preventive care are crucial, as poor oral hygiene can lead to infections, pain and difficulties in eating, speaking and learning. Therefore this review article addresses these challenges and offers strategies for effective periodontal care and treatment.

## Periodontal tissue in pediatric patients:

Periodontal tissues are in a continuous state of development, influencing their structure and function. The gingiva in children is typically more vascular and less keratinized, giving it a reddish hue and a softer, more rounded appearance at the gingival margin. This makes the gingiva more susceptible to inflammation and infection if proper oral hygiene is not maintained. The periodontal ligament in children is wider and more elastic, accommodating the growth and shifting of teeth as they erupt and settle into their permanent positions. This elasticity and width facilitate necessary adjustments during tooth development [6]. The alveolar bone in pediatric patients is more porous and less mineralized than in adults, providing the flexibility needed for the growth and eruption of teeth. Although this characteristic benefits dental development, it can also make the bone more vulnerable to infections and diseases if oral hygiene is neglected. Common periodontal issues in children include gingivitis, often resulting from poor oral hygiene and plaque accumulation [6, 7].

## Periodontal tissue changes in the elderly:

In elderly patients, periodontal tissues undergo significant changes due to the natural aging process, increasing susceptibility to periodontal diseases. Gingival recession, where the gum tissue pulls away from the teeth, exposes root surfaces and can lead to increased sensitivity and a higher risk of root caries [8]. Gingival tissue also becomes more fibrotic and less vascular with age, reducing its ability to repair and regenerate after injury or infection. Additionally, the periodontal ligament thins with age, losing some of its elasticity, making teeth more prone to mobility and loss [9]. The alveolar bone in elderly individuals typically experiences a decrease in density and mass, a process known as alveolar bone resorption. This condition can be exacerbated by long-term periodontal disease, leading to further tooth loss and difficulties with dental prosthetics, such as dentures. Changes in salivary flow and composition, often due to medications and systemic health conditions common in older adults, can also impact periodontal health by reducing the natural protective effects of saliva. Consequently, elderly patients are at a higher risk for severe forms of periodontitis [9, 10].

## Historical studies on periodontal disease in pediatric and geriatric populations:

Early studies on periodontal disease prevalence in India revealed high rates among various populations. Greene's study, using the Russell Periodontal Index (1956), examined a low socio-economic school population and found that 97% of 11-17 year-olds exhibited visible signs of periodontal disease, although less than 2% had periodontal pockets. Sanjana *et al.* reported that 83.2% of Bombay residents had signs of periodontal disease [2]. Despite these findings, national-level studies focusing on the geriatric population were very few. Generally, periodontitis prevalence increases with age. Dye and Selwitz identified that older age (≥40 years), lower education levels, smoking; male gender and non-Hispanic black ethnicity were linked to more severe periodontitis. They also observed a synergistic effect between aging and smoking in exacerbating risk [11]. In Sweden, Holm-Pedersen *et al.* studied individuals aged 80 and above, finding that 50.5% of those with teeth had severe periodontitis [12]. Similarly, a Finnish study on individuals aged 76-86 found that 46.2% were edentulous and among those with teeth, 11% had sites with probing depths of 6mm or more, indicating a significant need for periodontal treatment. Additional studies globally have reinforced these findings, highlighting the impact of socioeconomic factors, education, access to dental care, diet and oral hygiene practices on periodontal health among the elderly [13]. Research on pediatric periodontitis has focused on aggressive periodontitis, a less common but more severe and rapidly progressing form of the disease in children. Studies have linked this condition to genetic factors and systemic conditions, necessitating specialized care and early intervention. Recent research continues to explore the prevalence and risk factors of pediatric periodontitis, emphasizing the importance of early diagnosis, regular dental check-ups and education in fostering good oral hygiene habits from a young age [12, 13].

## Diagnosis of periodontal disease in geriatric and pediatric populations:

Diagnosing periodontal disease in geriatric and pediatric populations requires a nuanced approach tailored to their unique physiological and clinical characteristics. In pediatric patients, diagnosis often involves identifying early signs of gingivitis and aggressive periodontitis, which may be associated with genetic factors or systemic conditions. Pediatric dentists perform clinical examinations to assess gum inflammation, bleeding and pocket depths, supplemented by radiographs to detect bone loss and other structural anomalies. Early diagnosis is crucial to prevent the rapid progression of the disease, which can severely impact a child's oral and overall health [14]. Conversely, diagnosing periodontal disease in geriatric patients involves a comprehensive assessment of both oral and systemic health. Older adults are more likely to present with complex medical histories, including chronic diseases and medications that can affect periodontal health. Clinicians conduct thorough clinical examinations to evaluate gum recession, pocket depths, tooth mobility and attachment loss. Radiographs and advanced imaging techniques are employed to assess the extent of bone loss. Additionally, geriatric diagnosis must consider factors such as reduced manual dexterity, which can affect oral hygiene practices and the presence of dental prostheses. Effective diagnosis in both populations relies on early detection and a multidisciplinary approach to manage and mitigate the impact of periodontal disease [15, 16].

## Periodontal conditions in pediatric and geriatric populations:

The American Academy of Pediatric Dentistry recommends incorporating periodontal assessments into routine dental visits for children and adolescents. Plaque detection using disclosing agents helps identify problem areas, guiding oral hygiene practices for children and their parents. Routine screening methods, such as the Community Monitoring primary tooth loss and bone loss on radiographs are important indicators of periodontal disease. Developing screening tools for use by physicians and paediatricians is crucial, especially for identifying issues in children who may not regularly visit a dentist [17, 18]. Dental trauma can significantly impact the periodontium, leading to root resorption, ankylosis and alveolar bone loss. Healing varies based on the type of injury and stem-cell activity in the affected area. Recent advancements in understanding stem cells in periodontal tissues have improved management strategies, especially for immature permanent teeth. Effective treatment depends on the nature of the trauma and the regenerative potential of the tissues involved [19]. Studies have shown that obese children often exhibit more gingival inflammation, periodontal pockets and higher levels of inflammatory markers compared to their normal-weight peers. Obesity also affects salivary flow and increases plaque levels, potentially exacerbating periodontal conditions [20]. Children with enamel defects, such as molar incisor hypomineralization, amelogenesis imperfect or dentinogenesis imperfecta, often struggle with effective oral hygiene due to tooth sensitivity and increased fracture risk. Early surgical management and restorative treatments, such as composite restorations and crowns, are essential [21]. Measures like desensitizing toothpastes, soft brushes and fluoride sealants can improve oral hygiene and treatment outcomes. Addressing other anomalies, such as fused or supernumerary teeth, with combined pediatric dental and periodontal care can enhance long-term oral health [20, 21].

## Considerations for treatment planning in elderly and pediatric dental care:

When planning dental treatment for elderly individuals, a variety of factors must be considered to ensure effective and appropriate care. The OSCAR approach-Oral and dental needs, Systemic factors, Capability, Autonomy and Reality-is a comprehensive method that evaluates medical, dental, pharmacological, functional, ethical and financial aspects of dental management for older adults. Additionally, the "Clinical Oral Disorders in Elders" (CODE) index, developed by Macentee and Wyatt, assesses 27 clinical disorders across five major areas: mucosal health, jaw movements, dentures, teeth and peri-odontium and mucosal health. Combined with a psychosocial index, it provides a detailed indicator of oral dysfunction in the elderly [22]. Developing a tailored treatment plan is crucial for achieving optimal outcomes, particularly considering the extent of tissue destruction. Treatment plans often involve managing disease progression through non-surgical or surgical methods, along with lifestyle and dietary modifications to improve overall health and preserve natural dentition. For chronic periodontitis, treatment emphasizes mechanical debridement, including scaling and root planning, to remove plaque and calculus above and below the gum line [23]. Non-surgical interventions, such as scaling and root planning, are commonly used to manage various forms of periodontitis. For isolated pockets deeper than 5mm, local drug delivery systems like tetracycline fibers, chlorhexidine chips and minocycline microspheres have proven beneficial. In cases of more advanced periodontitis with pocket depths exceeding 7mm, subgingival irrigation systems can be effective adjuncts to scaling and root planning [24].

Surgical treatments are often necessary for moderate to advanced periodontitis, particularly after non-surgical approaches. These include soft tissue surgeries, osseous resective procedures and regenerative treatments. Minimally invasive periodontal therapies are increasingly preferred due to better healing and patient compliance. Periodontal surgery can address residual pockets or restore tooth substance in cases of fractures and crown/root caries. Resective osseous therapy aims to eliminate pathological pockets by reducing soft tissue depth and re-modelling supporting bone, while periodontal regeneration focuses on rebuilding lost periodontal tissue. Plastic surgery is used to correct soft tissue deformities, such as gingival recession, which is common in the elderly due to bone loss [23, 24]. Maintenance therapy is crucial for managing gingival recession in the elderly, which can cause sensitivity and tooth mobility, potentially leading to tooth loss. Minimally invasive management, including altering oral hygiene habits, prescribing home care devices for interdental cleaning and splinting mobile teeth, is often recommended. Regular dental visits and tooth retention are linked to improved quality of life for the elderly, as tooth loss can impair chewing efficiency, dietary choices, speech and psychological well-being [25]. Xerostomia frequently encountered in the elderly, increases the risk for dental caries, while increased attrition due to wear and tear necessitates restorative procedures and root canal therapies. Addressing these issues with appropriate treatment strategies can significantly enhance oral health and overall quality of life for elderly patients [26]. In pediatric dental care, treatment planning begins with non-surgical approaches such as scaling and root planning, along with antimicrobial treatments if necessary. These methods help control bacterial infection and reduce inflammation. For more severe cases, surgical interventions may be required. Treatment options should be carefully selected based on the child's age, cooperation and the severity of periodontal disease. Advances in pediatric dentistry, including fluoride varnishes, dental sealants, digital radiography and laser treatments, have markedly improved the quality and comfort of dental care for children [24, 25-26].

## Strategies for improving periodontal health in pediatric and geriatric populations:

To enhance periodontal health across different age groups, targeted strategies are essential. For the elderly, a comprehensive approach includes regular, thorough assessments that consider overall health, medications and individual needs, forming personalized care plans to address unique challenges. Interdisciplinary collaboration between dental professionals, primary care physicians and geriatric specialists ensures a holistic approach, particularly important for managing comorbid conditions impacting periodontal health. Education and support are vital; providing practical tips for maintaining oral hygiene and utilizing tools like electric toothbrushes can significantly aid those with physical limitations. Enhancing access to care through community programs, mobile dental clinics and telehealth services addresses socioeconomic barriers, while advocating for insurance coverage for elderly dental care at policy levels is crucial [24]. For pediatric patients, the dynamic nature of dental development presents unique challenges. The transition from primary to permanent teeth, along with habits like thumb-sucking, can affect periodontal health. Young children often struggle with effective oral hygiene due to limited cognitive and motor skills and dietary factors such as high sugar consumption exacerbate risks. Socioeconomic disparities further contribute to higher rates of periodontal issues among low-income families. Strategies to improve pediatric periodontal health include early education and intervention, with schools and paediatricians playing key roles in instilling lifelong oral health habits. Parental involvement is critical; educating parents on proper oral care techniques and the impact of diet on oral health can ensure effective home care. Expanding access to preventive services through community programs and school-based clinics helps bridge gaps for underserved populations. Behavioural approaches, such as using positive reinforcement and engaging tools like flavoured toothpaste and educational apps, can make oral hygiene routines more appealing to children, fostering better dental habits from an early age [23, 24- 26]. Nonetheless, it should be noted that periodontal health related challenges among geriatric and pediatric patients have also been reported recently [27].

## Conclusion:

Maintaining periodontal health in the elderly and children requires addressing their unique challenges. For the elderly, this includes managing comorbidities, cognitive and physical limitations and socioeconomic barriers. In pediatric patients, early education, parental involvement and access to preventive care are key to improving outcomes.
